# Synuclein Disorder‐Related Genetic Determinants of Mild Behavioural Impairment in a Pre‐Clinical Community Cohort

**DOI:** 10.1002/gps.70189

**Published:** 2026-03-19

**Authors:** Millie Sander‐Long, Byron Creese, Anne Corbett, Ivana Rosenzweig, Jeffrey Cummings, Clive Ballard

**Affiliations:** ^1^ Faculty of Life Sciences College of Medicine & Health University of Exeter Exeter UK; ^2^ Department of Life Sciences Brunel University London London UK; ^3^ Sleep Disorders Centre Guy's and St Thomas' NHS Foundation Trust London UK; ^4^ Department of Brain Health Chambers‐Grundy Center for Transformative Neuroscience School of Integrated Health Sciences University of Nevada Las Vegas Nevada USA

## Abstract

**Background:**

The *GBA* variant confers increased risk of synuclein disorders but it is unclear what impact it has in pre‐clinical groups. This study aimed to identify early psychiatric and cognitive manifestations amongst pre‐clinical *GBA* carriers in a community cohort.

**Method:**

This study used data from the PROTECT‐UK cohort to compare 388 *GBA* carriers (N370S, E326K and T369M) without Parkinson's disease to age‐matched controls. Neuropsychiatric symptoms (NPS) were measured with the Mild Behaviour Impairment Checklist, and cognition was measured using computerised neuropsychology.

**Results:**

**Results:** *GBA* carriers over 70 had significantly increased NPS compared with controls (*z* = 2.13, *p* = 0.03). There was no difference between carriers and non‐carriers in younger individuals but a sub‐group comparison in the overall cohort showed that NPS were more severe in quartile four (Q4) of carriers compared to Q4 of controls (*z* = 2.39, *p* = 0.017), indicating an increase in NPS in this sub‐group across a broader age range. No differences in cognition were seen.

**Discussion:**

These findings suggest that NPS may be an early clinical manifestation of emerging synucleinopathy amongst individuals prior to diagnosis.

## Key Points

- This study examines neuropsychiatric symptoms in pre‐clinical carriers of GBA variants
- GBA carriers over 70 years old show significantly more pre‐clinical neuropsychiatric symptoms, and carrier status is linked to severity of symptoms
- Neuropsychiatric symptoms may be an early clinical manifestation of emerging synucleinopathy amongst pre‐clinical carriers, raising the potential for early risk profiling based on genetic status

## 1 Introduction

Parkinson's Disease (PD) and related synuclein dementias such as Dementia with Lewy bodies (DLB) and Parkinson's disease dementia (PDD) are often associated with neuropsychiatric symptoms (NPS) such as depression and psychosis [1]. These symptoms present challenges for treatment and care and are the cause of considerable distress. Other core clinical features include dementia with prominent deficits in attention and executive function, parkinsonism (motor impairment) and fluctuating cognition [2, 3, 4].

Variants of the glucosylceramidase beta 1 (*GBA1*) gene are present in just under 1% of the general population, with a significantly higher frequency in the Ashkenazi Jewish population [3, 5]. The most common *GBA1* variants are E326K, N307S, L444P, T369M, and RecNil [3]. GBA1 is associated with an 8‐10‐fold increased risk of PD, DLB and PDD [6, 7] and is present in 10%–15% of individuals with PD [8]. The symptom cluster in GBA carriers with PD often includes early presentation of visual hallucinations and other psychotic symptoms combined with depression [9]. There are currently no specific studies focusing on NPS at this very early pre‐clinical stage. In the general population, research has suggested that mood symptoms, irritability and apathy are the most common emerging NPS in people with mild behavioural impairment, but it is likely that depression and pre‐psychotic symptoms may be more common in GBA carriers. *GBA1* carriers with PD also present with more severe deficits in working memory and executive functioning, rapid cognitive decline and a specific association with Lewy Body pathology [9]. The age of symptom onset is also more than 5 years earlier in DLB patients carrying *GBA1* variants compared with non‐carriers [5, 9, 10, 11, 12].

The presentation of synuclein disorders is also dependent on the *GBA1* variant present. *GBA1* variants can be classified as either ‘mild’ or ‘severe’ variants, based on the subtype of Gaucher's disease (GD) that they cause. GD type 1 (non‐neuropathic) is caused by mild variants such as E326K, whilest GD types 2 (acute neuropathic) and type 3 (chronic neuropathic) are caused by severe variants such as L444P [13, 14]. Whilst both mild and severe variants are associated with synucleinopathies such as PD, cognitive decline including progression to dementia, motor dysfunction, younger age of onset, and increased risk of mortality are more significantly associated with severe variants, with mild variants only presenting a moderately increased risk of such symptoms [14, 15]. The likelihood of presenting with a synuclein disorder is also greater among those with severe variants, with Gan‐Or et alreporting the frequency of PD to be 4‐fold among those with severe variants compared with those harbouring mild variants (with 4.4% and 0.9% of carriers presenting PD, respectively) [13].

Although the *GBA1* variants are therefore well established as a significant risk factor for PD, LBD and PDD, the impact of the GBA variants in individuals without overt clinical symptoms is less well established. One small study in 28 patients indicated a subtle early cognitive impairment in GBA carriers who were free of PD symptoms [16, 17] but there was no assessment of NPS. Better understanding of pre‐clinical presentations could provide novel opportunities for precision approaches to prevention and treatment amongst *GBA1* carriers who are at significantly increased risk of synuclein disorders and related dementias. Given the indications that GBA variants are associated with early presentation of NPS and prominent executive and attention dysfunction, this study compared the presence of NPS (using the mild behavioural impairment (MBI) framework) and impairments in executive and attentional performance between *GBA1* variant carriers and non‐carriers in community‐based participants of the PROTECT‐UK ageing research cohort.

## 2 Methods

### 2.1 Participant Recruitment

Participants were recruited via the PROTECT‐UK study (www.protectstudy.org.uk; Research Ethics Committee reference number 13/LO/1578). Inclusion criteria for PROTECT‐UK are (1) being aged 50 or older at the time of collection for this analysis; (2) access to a computer and internet; and (3) no diagnosis of dementia. Participation in PROTECT‐UK is voluntary, with participants providing informed consent online at the time of enrolment. For this study, participants with an established diagnosis of PD were excluded.

### 2.2 Genotyping

DNA was collected via saliva kits. Long range phasing and sequence variant calling was performed jointly for multiple cohorts where 50,839 have been subjected to Whole Genome Sequencing and 1,041,174 chip‐typed as previously described [18]. All were sequenced to a minimum depth of 20x. Variant calling was performed using GraphTyper18 and sequence variants imputed into chip‐typed Norwegians, Swedes and Danes. The three GBA variants examined were N370S, E326K and T369M.

### 2.3 Data Sampling

As part of the PROTECT study, participants complete annual computerised assessment of cognition in addition to self‐report questionnaires. For this study, data were a subset including information only from individuals for whom genotyping data were available. An age‐ and sex‐matched dataset was created to enable comparison of any GBA carrier with non‐carriers. Age groups were created by splitting the cohort into under‐70 and over 70 years old as an arbitrary threshold to examine impact in higher risk individuals based on age.

### 2.4 Mild Behaviour Inventory

Participants completed the Mild Behaviour Inventory Checklist (MBI) on the PROTECT‐UK study platform (MBI) [19]. The MBI‐C is a simple, easy to administer and well validated NPS rating scale comprising 34 questions that capture five domains (Interest/Motivation, Mood, Irritability, Impulse Control and Psychosis) that are categorised as mild, moderate and severe, with a total score > 8 indicating significant NPS [19, 20].

### 2.5 Cognitive Outcomes

Cognitive assessments were conducted on the PROTECT‐UK study platform using the PROTECT Cognitive Test System which uses well validated computerised neuropsychological assessments [21]. Data for this study were derived from five tests to capture executive function, attention, working memory and episodic memory. Executive function was measured using the Baddeley Verbal Reasoning test in which users are presented with a series of images containing a circle and a square and must select whether a statement describing the image is true or false. Attention was measured using the choice reaction time and digit vigilance tests. Choice reaction time involves responding to an on‐screen stimulus by selecting a target of the same colour, and digit vigilance involves responding every time a nominated digit appears on the screen in a scrolling series of digits. Both tests capture reaction time and accuracy as measures of attention. Working memory was measured using Paired Associate Learning in which the user must remember and correctly identify the location of a symbol in a grid of hidden boxes. Episodic memory was measured using the Delayed Picture Recognition test in which users are shown pictures of everyday objects. After a 30‐min delay, they must then identify the objects they have seen previously when mixed with similar objects presented in sequence. These tests are validated for sensitivity to cognitive and functional status and change and are widely applied in research settings for sensitive assessment of cognitive performance [21].

### 2.6 Analysis

Control participants were matched with *GBA1* variant carriers based on age and sex. The primary analysis was focussed on the influence of *GBA1* variants on NPS and cognition, comparing GBA carriers > 70 with age matched controls. Further analyses compared GBA carriers < 70 with age matched controls, and for NPS compared individuals with the highest MBI scores (Quartile 4 (Q4)) amongst GBA carriers with Q4 of the control group across the full age range to determine whether there was an impact of GBA in the subgroup with the most severe NPS. Analyses were conducted using regression models (Poission, (zero‐inflated) negative binomial, or Gamma depending on distribution of the data). All evaluations were undertaken in *R* Studio (version 4.3).

## 3 Results

### 3.1 Cohort Characteristics

Among the PROTECT‐UK database, genotyping information was available 9169 individuals. Of these, 401 individuals carried variants of the *GBA1* gene. Variants were E326K, N307S and T369M, all of which represent medium level of risk. After removing any individuals with incomplete data, 388 GBA carriers with cognitive data remained, who were subsequently age and sex matched with 388 healthy controls. A total of 631 (*n* = 317 carriers, and *n* = 314 controls) observations were available for MBI‐C. The analysis cohort characteristics are shown in Table 1. No participants reported any psychiatric disorders. 7% of GBA carriers and 4% of controls reported a diagnosis of depression.

**TABLE 1:** GBA1 carrier cohort characteristics.

		GBA carriers (*n* = 388)	Non‐carriers (*n* = 388)
Sex	Male	89 (22.9%)	89 (22.9%)
	Female	299 (77.1%)	299 (77.1%)
Age group	50–59	169 (43.56%)	169 (43.56%)
	60–69	176 (45.36%)	176 (45.36%)
	70–79	40 (10.31%)	40 (10.31%)
	80–89	3 (0.77%)	3 (0.77%)
Education level	1—(Post) secondary education (GCSE/O‐levels, college, A‐levels, or similar)	96 (24.74%)	109 (28.09%)
	2—Vocational qualification (diploma, certificate, BTEC, NVQ 4 and above, or similar)	78 (20.1%)	69 (17.78%)
	3—Higher education (undergraduate/postgraduate degree, doctorate, etc.)	214 (55.15%)	210 (54.12%)

### 3.2 Neuropsychiatric Symptoms

**FIGURE 1:**
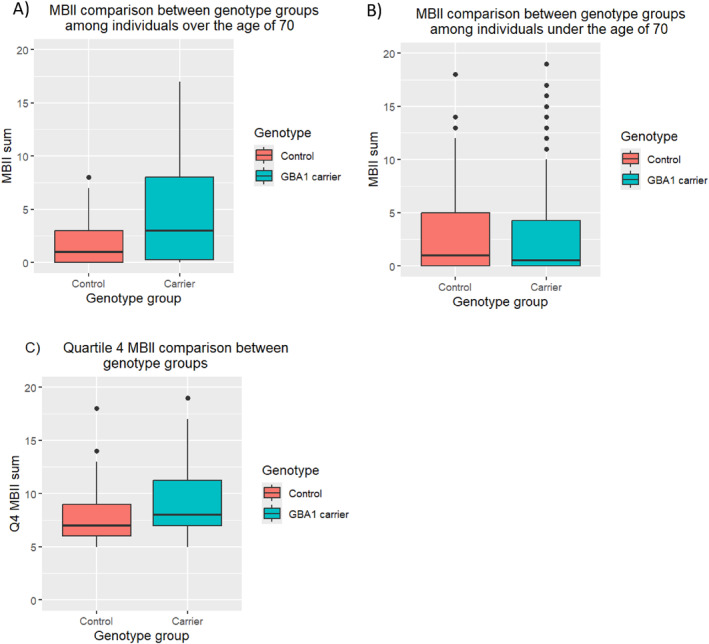
Comparison of MBI‐I scores between GBA carriers and non‐carriers in individuals over (A) and under (B) 70 and in quartile 4 of MBI scores (C).

Amongst individuals over the age of 70, there was an overall significant difference in total MBI score between *GBA1* carriers and controls (*b* = 0.59, SE = 0.28, *z* value = 2.13, *p* = 0.03) (Figure 1A). GBA1 carriers over the age of 70 were also significantly more likely to score an MBI score of eight or above compared to controls (OR = 6.22, *p* = 0.03), indicating a substantial increase in significant overall NPS (Figure 1B). In contrast, amongst individuals under the age of 70, no significant difference in total MBI score was observed between *GBA1* variant carriers and controls < 70 (*b =* 0.09, SE = 0.14, *z* value = 0.69, *p* = 0.49). In comparisons of different MBI domains, among individuals over the age of 70, genotype was significantly associated with both activity interest (*b* = 1.35, SE = 0.65, *z* value = 2.08, *p* = 0.04) and emotion (*b* = 0.81, SE = 0.36, *z* value = 2.25, *p* = 0.02) domains. For the activity interest domain, 34.62% of *GBA1* carriers were affected on at least one outcome measure, compared with 13.33% of controls. For the emotion domain, these percentages are 65.38% and 36.67% for *GBA1* carriers and controls, respectively. No significant association was found between genotype and the actions, attitudes, or thoughts (psychosis) domains, with the percentage of individuals reporting any domain symptom being 50% and 50% for actions, 15.38% and 13.33% for attitudes, and 0% and 3.33% for thoughts among *GBA1* carriers and controls, respectively.

Among individuals under the age of 70, no significant association between *GBA1* genotype and any of the five MBI domains were observed.

Although the risk of synuclein disorders is substantially increased amongst GBA carriers, the mutations are not fully penetrant and only a minority of individuals with the risk variants develop these conditions. Therefore, further analyses were undertaken across both age groups (individuals > 70 and individuals < 70) to compare individuals in *h* = the highest quartile MBI score (Q4) between the GBA group and the control group. Analysis indicated a significant difference in MBI total score (*b* = 0.16, SE = 0.07, *z* value = 2.4, *p* = 0.02), with carriers scoring significantly higher than controls (Figure 1C).

There were no significant differences in MBI scores between the different GBA variants (Supporting Information S1: Table 1).

### 3.3 Cognitive Outcomes

Amongst individuals > 70 no significant difference was observed in any cognitive variable in GBA carriers compared with controls (VIGACC *p* = 0.1, PALTOT *p* = 0.65, DPICOACC *p* = 0.67, VERBTOT *p* = 0.3, RTS2_RTM *p* = 0.87) (Supporting Information S1: Table 2). Similarly, no significant differences were observed between *GBA1* variant carriers and controls among those under the age of 70 (VIGACC *p* = 0.53, PALTOT *p* = 0.85, DPICOACC *p* = 0.26, VERBTOT *p* = 0.46, RTS2_RTM *p* = 0.45), The quartile of GBA carriers with the lowest level of performance on the cognitive tests(Q4) was compared with Q4 of the control group across all age groups (> 70 and < 70) and again no significant difference in any outcome measure was observed among carriers compared with controls (VIGACC *p* = 0.32, PALTOT *p* = 0.99, DPICOACC *p* = 0.39, VERBTOT *p* = 0.73, RTS2_RTM *p* = 0.52). There were no significant differences on any of the tests between the different GBA variants or in individuals with elevated (> 8) MBI scores.

## 4 Discussion

This study represents the first large‐scale investigation into neuropsychiatric and cognitive features in community‐based cognitively unimpaired individuals carrying GBA1 variants, in the absence of PD or dementia diagnoses. The cohort included 388 *GBA* carriers from the PROTECT cohort. Significant differences in NPS were evident between GBA carriers and controls in people over the age of 70. In contrast, no significant differences were seen in NPS comparing GBA carriers and controls under the age of 70. Sub‐group analysis of individuals within the highest quartile of MBI score also demonstrated a significant increase in MBI amongst GBA carriers, suggesting the possibility of a subset of individuals in whom prodromal synucleinopathy may manifest initially through neuropsychiatric changes. The evaluation also compared cognition but found no significant indications of prodromal cognitive impairment in GBA carriers, even in people over 70. Together, these results indicate that NPS may be an early and important indicator of *GBA1*‐associated impairment that precedes cognitive impairment and may therefore serve as an important screening tool among these individuals. These findings support growing evidence that NPS may constitute one of the earliest clinical indicators of underlying neurodegenerative processes, including amongst genetically at‐risk populations [22, 23].

This study provides the first evaluation of a large cohort of individuals carrying *a GBA* allele without the presence of PD and reports a robust evaluation of NPS and cognition. Limitations include the cross‐sectional design and the absence of neuroimaging or biomarker correlates. Thus, further studies to examine prodromal PD symptoms, including autonomic dysfunction, rapid eye movement (REM) sleep behavioural disturbance and any related biomarker changes will be an important extension of this work. This will create important precision medicine opportunities. For example, mice carrying *GBA* variants exhibit early cholinergic deficits [24], and emerging safer muscarinic agonists [25, 26, 27] with potential actions on cognition and psychosis may be an excellent candidate therapy. Realizing these opportunities will require the identification of GBA1 carriers at highest risk of phenoconversion, alongside longitudinal tracking of prodromal symptoms and integration with multimodal biomarker data.

## Conflicts of Interest

C.B. has received consulting fees from Acadia pharmaceutical company, AARP, Addex pharmaceutical company, Eli Lily, Enterin pharmaceutical company, GWPharm, H.Lundbeck pharmaceutical company, Novartis pharmaceutical company, Janssen Pharmaceuticals, Johnson and Johnson pharmaceuticals, Novo Nordisk pharmaceutical company, Orion Corp pharmaceutical company, Otsuka America Pharm Inc, Sunovion Pharm. Inc, Suven pharmaceutical company, Roche pharmaceutical company, Biogen pharmaceutical company, Synexus clinical research organization and tauX pharmaceutical company and research funding from Synexus clinical research organization, Roche pharmaceutical company, Novo Nordisk pharmaceutical company and Novartis pharmaceutical company. J.C. has provided consultation to Acadia, Acumen, ALZpath, Annovis, Artery, Axsome, Biogen, Biohaven, Bristol‐Myers Squib, Cervomed, Eisai, Fosun, GAP Foundation, Green Valley, Hummingbird Diagnostics. IGC, Janssen, Julius Clinical, Kinoxis, Lighthouse, Lilly, Lundbeck, LSP/eqt, Merck, MoCA Cognition, Novo Nordisk, NSC Therapeutics, Optoceutics, Otsuka, Praxis, ReMYND, Roche, Scottish Brain Sciences, Signant Health, Simcere, sinaptica, T‐Neuro, TrueBinding, and Vaxxinity pharmaceutical, assessment, and investment companies. J.C. is co‐founder of CNS Innovations and Mangrove Therapeutics. A.C. discloses financial relationships with Suven and Janssen pharmaceutical companies for consultancy work. Other authors have no conflicts to disclose.

## Supporting information


Supporting Information S1

## Data Availability

The data that support the findings of this study are available from the corresponding author upon reasonable request.
