# Supplementary material for: Synuclein Disorder‐Related Genetic Determinants of Mild Behavioural Impairment in a Pre‐Clinical Community Cohort
Source: Int J Geriatr Psychiatry. 2026 Mar 19;41(3):e70189. doi: 10.1002/gps.70189 (PMC13001855; doi:10.1002/gps.70189)
Supplement: Supplementary file 1 — Supporting Information S1 [file GPS-41-e70189-s001.docx]

**Supplementary Table 1: Comparison of MBI scores in different GBA variants in participants >70 and <70**

| **Comparison** | **Test estimate** | **Standard error** | **t value** | **p value** |
| --- | --- | --- | --- | --- |
| **MBII Under 70s** | | | | |
| **E326K** | 0.21 | 0.15 | 1.36 | 0.18 |
| **N307S** | -0.25 | 0.37 | -0.67 | 0.51 |
| **T369M** | -0.09 | 0.2 | -0.45 | 0.66 |
| **MBII Over 70s** | | | | |
| **E326K** | 0.38 | 0.33 | 1.15 | 0.25 |
| **N307S** | 0.75 | 0.43 | 1.74 | 0.08 |
| **T369M** | 0.28 | 0.36 | 0.78 | 0.43 |

**Supplementary Table 2:** **Cognitive outcomes for GBA carriers in the entire study cohort, individuals >70 and <70**

| **Comparison** | **Test estimate** | **Standard error** | **t value** | **p value** |
| --- | --- | --- | --- | --- |
| **Entire cohort** | | | | |
| **VIGACC** | -0.16 | 0.16 | -0.99 | 0.32 |
| **PALTOT** | -0.0013 | 0.072 | -0.017 | 0.99 |
| **DPICOACC** | -0.07 | 0.08 | -0.87 | 0.39 |
| **VERBTOT** | -0.22 | 0.64 | -0.35 | 0.73 |
| **RTS2 RTM** | -3.08 | 4.78 | -0.64 | 0.52 |
| **Under 70s** | | | | |
| **VIGACC** | -0.11 | 0.18 | -0.63 | 0.53 |
| **PALTOT** | 0.014 | 0.073 | 0.19 | 0.85 |
| **DPICOACC** | -0.1 | 0.087 | -1.13 | 0.26 |
| **VERBTOT** | -0.5 | 0.67 | -0.74 | 0.46 |
| **RTS2 RTM** | -3.82 | 5.07 | -0.75 | 0.45 |
| **Over 70s** | | | | |
| **VIGACC** | -0.45 | 0.27 | -1.65 | 0.1 |
| **PALTOT** | -0.12 | 0.26 | -0.46 | 0.65 |
| **DPICOACC** | 0.12 | 0.28 | 0.43 | 0.67 |
| **VERBTOT** | 1.95 | 1.88 | 1.04 | 0.3 |
| **RTS2 RTM** | 2.08 | 13.08 | 0.16 | 0.87 |
| **Entire cohort, quartile 4** | | | | |
| **VIGACC** | -3.813e-14 | 1.661e-13 | -2.300e-01 | 0.82 |
| **PALTOT** | -0.02 | 0.09 | -0.14 | 0.89 |
| **DPICOACC** | -3.27 | 1.58 | -2.07 | 0.04 * |
| **VERBTOT** | -0.17 | 0.6 | -0.29 | 0.78 |
| **RTS2 RTM** | -9.08 | 7.34 | -1.24 | 0.22 |
